# Altered anterior visual system development following early monocular enucleation^☆^

**DOI:** 10.1016/j.nicl.2013.10.014

**Published:** 2013-11-01

**Authors:** Krista R. Kelly, Larissa McKetton, Keith A. Schneider, Brenda L. Gallie, Jennifer K.E. Steeves

**Affiliations:** a Department of Psychology, York University, Toronto, Canada; b Centre for Vision Research, York University, Toronto, Canada; c Department of Biology, York University, Toronto, Canada; d Department of Ophthalmology and Visual Sciences, The Hospital for Sick Children, Toronto, Canada; e Department of Ophthalmology, University of Toronto, Toronto, Canada; f Princess Margaret Cancer Centre, University Health Network, Toronto, Canada

**Keywords:** Retinoblastoma, Monocular enucleation, Anterior visual system development, Optic chiasm, Lateral geniculate nucleus

## Abstract

**Purpose:**

Retinoblastoma is a rare eye cancer that generally occurs before 5 years of age and often results in enucleation (surgical removal) of the cancerous eye. In the present study, we sought to determine the consequences of early monocular enucleation on the morphological development of the anterior visual pathway including the optic chiasm and lateral geniculate nucleus.

**Methods:**

A group of adults who had one eye enucleated early in life due to retinoblastoma was compared to binocularly intact controls. Although structural changes have previously been reported in late enucleation, we also collected data from one late enucleated participant to compare to our early enucleated participants. Measurements of the optic nerves, optic chiasm, optic tracts and lateral geniculate nuclei were evaluated from T_1_ weighted and proton density weighted images collected from each participant.

**Results:**

The early monocular enucleation group exhibited overall degeneration of the anterior visual system compared to controls. Surprisingly, however, optic tract diameter and geniculate volume decreases were less severe contralateral to the remaining eye. Consistent with previous research, the late enucleated participant showed no asymmetry and significantly larger volume decreases in both geniculate nuclei compared to controls.

**Conclusions:**

The novel finding of an asymmetry in morphology of the anterior visual system following long-term survival from early monocular enucleation indicates altered postnatal visual development. Possible mechanisms behind this altered development include recruitment of deafferented cells by crossing nasal fibres and/or geniculate cell retention via feedback from primary visual cortex. These data highlight the importance of balanced binocular input during postnatal maturation for typical anterior visual system morphology.

## Highlights

• Morphology of the anterior visual system in early monocular enucleation was assessed.
• Optic tract diameter, and optic chiasm and lateral geniculate volumes were decreased.
• A contralateral bias was observed for optic tract diameter and geniculate volume.
• One late enucleate exhibited severe geniculate volume decreases but no asymmetry.
• Altered visual development occurs to compensate for early enucleation.

## 1 Introduction

Retinoblastoma is a rare eye cancer that accounts for approximately 6% of all childhood cancers and generally occurs before 5 years of age (Broaddus et al., 2009a). The most frequent and effective treatment for unilateral retinoblastoma is monocular enucleation (surgical removal) of the cancerous eye, which, in turn, deprives the brain of one half of visual inputs. Due to the high survival rate (97%) (Broaddus et al., 2009b) and lack of balanced binocular input associated with retinoblastoma, it is crucial to study the consequences of the most effective treatment, monocular enucleation, on visual system development following long-term survival. In both animals and humans, postnatal monocular deprivation from lid suture, congenital cataracts, and strabismus negatively affects visual development (e.g. Wiesel and Hubel, 1963; Ellemberg et al., 2000; Barnes et al., 2010) given that, at birth, the visual system is not fully mature (reviewed in Daw, 2006). This finding highlights the importance of receiving binocular balanced input during the critical postnatal developmental period of the visual pathway.

**Fig. 1:**
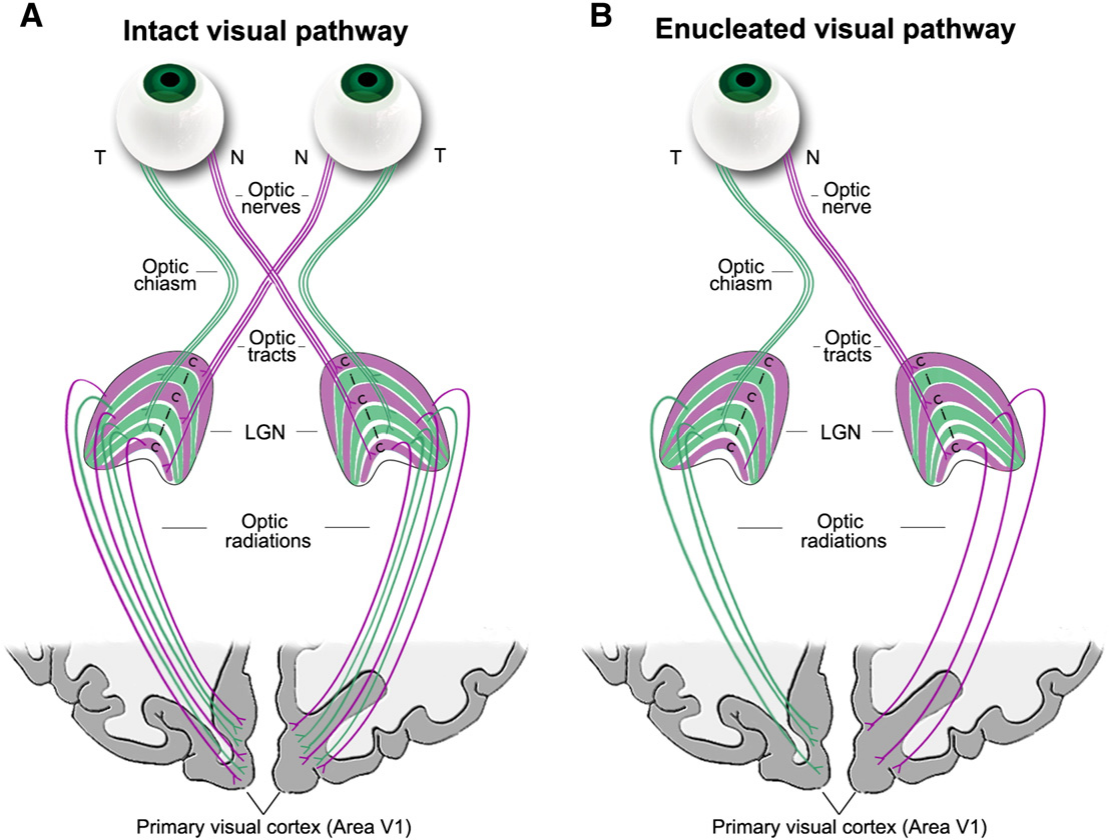
Schematic of the (A) binocularly intact and (B) monocularly enucleated visual pathways. (A) In the intact visual pathway, visual information from each eye travels through the optic nerve via the temporal (T: green) and nasal (N: purple) retinal fibres and converges at the optic chiasm. Uncrossed temporal fibres project to the ipsilateral LGN and crossed nasal fibres project to the contralateral LGN which receive information from each eye in separate layers. From here, the optic radiations carry information to primary visual cortex (cortical area V1). (B) In the monocularly enucleated visual pathway, the right eye is removed in this diagram and half of the inputs received by the LGN and visual cortex are deafferented.

Here, we focus on the morphological development of the anterior visual pathway in adults who have had one eye enucleated early in life due to retinoblastoma. In the primate visual pathway, retinal ganglion cell axons from each eye converge at the optic chiasm. Approximately half of these axons project ipsilaterally (uncrossed temporal fibres) while the other half project contralaterally (crossed nasal fibres) via the optic tracts to each lateral geniculate nucleus (LGN) (Fig. 1). The LGN is a subcortical thalamic visual relay station that receives information from each eye in segregated, eye-specific laminae. At birth, this structure is relatively adult-like in volume and laminar organization and is morphologically mature by 9 months of age; however, it continues to develop physiologically (de Courten and Garey, 1982; Garey and de Courten, 1983; Blakemore and Vital-Durand, 1986). Frontally placed eyes and thus an overlap of the visual fields in primates results in binocular vision, specifically stereopsis and binocular depth perception, that occurs at a cortical level in striate and extrastriate visual areas. Hemidecussation of retinal fibres allows binocular cells within these regions to receive information from both eyes about corresponding points in the binocular visual field (reviewed in Howard, 2002). Since monocular enucleation results in only one stream of visual information to the brain and thus a lack of binocularity within the cortex, development of the enucleated visual system is likely to be disrupted.

Behavioural consequences of early monocular enucleation in adult humans are well documented. In general, this population exhibits intact or enhanced spatial vision, such as contrast sensitivity (Nicholas et al., 1996; Steeves et al., 2004; Kelly et al., 2013a), but mildly impaired motion perception, such as speed discrimination (Kelly et al., 2013a; reviewed in Steeves et al., 2008; Kelly et al., 2013b). Few researchers have had the opportunity to investigate morphological changes in the enucleated human visual pathway. Morphological changes have been relatively well established in animal models of enucleation, although most of these models are from species with more laterally placed eyes and therefore predominantly crossed retinal fibres. These studies show an increase in crossed fibres of the remaining eye at the expense of uncrossed fibres in enucleated mice, ferrets, and rabbits (Grigonis et al., 1986; Godement et al., 1987; Guillery, 1989). In enucleated rabbits, LGN size contralateral to the enucleated eye is reduced (Khan, 2005). While these studies demonstrate changes as a result of early eye enucleation, it is difficult to extrapolate these results to the primate visual system given the fundamental differences in the species' visual systems: rodents exhibit a crossing over of the majority of retinal fibres and a lack of binocular disparity and stereopsis while primates exhibit hemidecussation of retinal fibres and no lack of binocular disparity and stereopsis (Jeffery et al., 1981; Provis and Watson, 1981; Chalupa and Lia, 1991; Neveu et al., 2006). In non-human primates, geniculate laminae fail to segregate (Rakic, 1981), and deafferented and non-deafferented cells shrink following enucleation (Matthews, 1964; Hubel et al., 1977; Hendrickson and Tigges, 1985; Sloper et al., 1987). Yet, it is also difficult to generalize non-human primate studies to early enucleated humans since some non-human primate studies focus on enucleation in adulthood (e.g. Hendrickson and Tigges, 1985) and all studies only report consequences following a relatively short survival period in late or early enucleation (Matthews, 1964; Hubel et al., 1977; Rakic, 1981; Hendrickson and Tigges, 1985; Sloper et al., 1987).

Only a handful of studies have focused on morphological changes following early monocular enucleation in humans. For example, reduced optic chiasm width (Horton, 1997), degenerated optic tracts, and transneuronal degeneration of deafferented geniculate cells (Goldby, 1957; Hickey and Guillery, 1979; Beatty et al., 1982) are reported years following enucleation. However, most studies have only reported brain changes in those who have lost one eye during adulthood when the developmental critical periods have long been surpassed. Moreover, these examinations were all completed postmortem. One study did examine the LGN of an adult who lost one eye at 6 years of age due to trauma and found transneuronal degeneration of layers associated with the enucleated eye (Hickey and Guillery, 1979), but this examination was only conducted on the left LGN and it is unknown how the right LGN was affected.

Our goal in the present study was to assess how early eye enucleation affects the maturation of the anterior visual system given that this system is not receiving information from the enucleated eye that is required during postnatal development. More specifically, we sought to examine these effects following long-term survival. To do so, we used noninvasive structural magnetic resonance imaging (MRI) to assess the optic nerves, optic chiasm, optic tracts, and LGN in adults who had one eye enucleated during infancy due to retinoblastoma and compared them to binocularly intact controls. Although previous research has reported on late enucleated participants and the structure of the anterior visual system, we had the opportunity to test one late enucleated participant and compare his results to the control and early enucleation groups. Consistent with early enucleated monkeys (Matthews, 1964; Rakic, 1981; Sloper et al., 1987) and late enucleated humans (Goldby, 1957; Beatty et al., 1982; Horton, 1997), we predict reductions in optic chiasm and LGN measures in the early enucleation group. This finding would suggest degeneration of cells in a system that relies on Hebbian synaptic learning for refinement during maturation. Alternatively, if early enucleation alters the complex development of the visual system rather than triggering cell loss, we predict a different pattern of results that would be present with early, but not late, enucleation. These data will help elucidate the role of balanced binocular vision during postnatal maturation in the developing anterior visual system.

## 2 Methods

### 2.1 Participants

#### 2.1.1 Early monocular enucleation (ME) group

The first phase of this study consisted of analyzing the morphology of the chiasm of twelve adults (6 males) who were former patients at The Hospital for Sick Children in Toronto and who had one eye enucleated due to retinoblastoma (cancer of the retina). Mean age (± SD) was 27.4 ± 11.9 years (range = 17–54 years) and mean age at enucleation (AAE) (± SD) was 18.7 ± 11.3 months (range = 4–48 months). Based on the size and position of the tumour under retinal examination prior to enucleation, it is estimated that typically the tumour would have lead to disrupted vision approximately 6 months prior to enucleation. Eight of these participants (3 males) also took part in the second phase of this study which consisted of analyzing LGN volume. Mean age (± SD) was 27.8 ± 9.7 years (range = 17–45 years) and mean AAE (± SD) was 15.0 ± 6.4 months (range = 4–26 months). All participants had normal or corrected-to-normal acuity as assessed by an EDTRS eye chart (Precision Vision™, La Salle, IL). Enucleated participants are regularly seen by their ophthalmologist. No known ocular abnormalities in the remaining eye, or neurological abnormalities in the brain, were reported. Table 1 lists individual patient histories.

#### 2.1.2 Late ME participant

We also tested one late ME participant (male, age = 65 years) who experienced trauma to the eye at 50 years of age that eventually resulted in a detached retina and subsequent eye enucleation approximately 6 years prior to testing (AAE = 708 months). All data from the late ME participant was analyzed separately from the early ME group to compare late versus early enucleation (see Table 1).

#### 2.1.3 Control group

Twenty-eight binocularly intact controls (15 males) took part in the chiasm phase of the study and were approximately age- and sex-matched to the early ME group. Mean age (± SD) was 29.9 ± 11.6 years (range = 18–60 years). Fifteen of these participants (8 males) also took part in the LGN phase and mean age (± SD) was 29.9 ± 11.2 years (range = 18–60 years). Participants had normal or corrected-to-normal acuity (Precision Vision™, La Salle, IL) and normal Titmus stereoacuity (Stereo Optical Co., Inc., Chicago, IL). Acuity and the Porta test (described in Durand and Gould, 1910) were used to assess eye dominance (Chiasm: 18 right-eye dominant; LGN: 11 right-eye dominant).

### 2.2 Data acquisition, processing, and measurements

This research followed the Declaration of Helsinki doctrine and was approved by the Research Ethics Boards of both The Hospital for Sick Children and York University. Informed consent was obtained from all participants prior to testing and after explanation of the nature and possible consequences of the study.

**Fig. 2:**
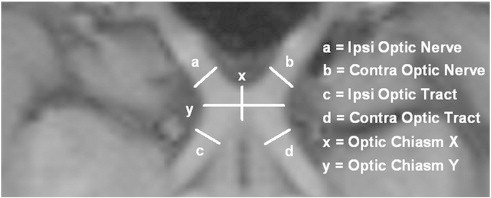
An axial image of a reformatted T_1_ weighted scan from a control participant displaying optic nerves (a) ipsilateral and (b) contralateral to the dominant eye, optic tracts (c) ipsilateral and (d) contralateral to the dominant eye, and optic chiasm widths in the (x) X and (y) Y planes.

All scans were acquired on a Siemens MAGNETOM Trio 3 T MRI scanner with a 32-channel head coil at the Sherman Health Sciences Research Centre, York University. A T_1_ weighted three-dimensional MPRAGE scan of the entire head was acquired sagitally with the following parameters: rapid gradient echo, 1 mm^3^ isotropic voxels, TR = 1900 ms, TE = 2.52 ms, 256 × 256 matrix, and flip angle = 9°. All T_1_ images were re-oriented on the MRI console (Syngo; Siemens, Munich, Germany) to ensure the optic nerves, optic chiasm, and optic tracts were observed in the same plane and a reformatted image with a 1 mm slice thickness was obtained parallel to the optic chiasm. Processing of these reformatted images was conducted using tools from the freely available FMRIB's Software Library (FSL; version 4.1.8) (http://www.fmrib.ox.ac.uk/fsl). Chiasm region-of-interest (ROI) masks were manually traced by three independent raters blind to group membership in FSLview. Chiasm masks from each rater were merged using fslmerge and a median mask was created using fslmaths that was comprised of voxels chosen in at least 2 of the 3 original masks. From the median mask, chiasm volumes were calculated using the fslstats tool. Several chiasm measurements (Fig. 2) were also taken by four independent raters blind to group membership using the OsiriX® length measurement tool, which computes selected distances using bicubic interpolation (Rosset et al., 2004): optic nerve diameter (a) ipsilateral and (b) contralateral to the dominant eye for controls and the remaining eye for ME participants, optic tract diameter (c) ipsilateral and (d) contralateral to the dominant/remaining eye, and optic chiasm width in the (x) X and (y) Y planes. Each rater took three measurements and the average of these measurements across raters was calculated per structure. To ensure whole brain volume was not a factor affecting our results, we also calculated whole brain volumes excluding the cerebellum and brain stem using volumetric segmentation performed with the FreeSurfer image analysis suite (http://surfer.nmr.mgh.harvard.edu/).

While T_1_ weighted images have good contrast between grey matter and white matter in the cortex, the contrast between the LGN and surrounding tissue is low, making it difficult to differentiate the LGN. Proton density (PD) weighted images have been shown to successively distinguish thalamic nuclei (Devlin et al., 2006). PD weighted images use a long repetition time and a short echo time to minimize T_1_ and T_2_ weightings, thus the tissue contrast is primarily dependent on the number of protons per unit tissue (i.e. proton density) (Jackson et al., 1997). A higher density of protons gives rise to brighter signals on the PD image, and vice versa. Following T_1_ acquisition, our participants underwent between 30 and 40 PD weighted scans each lasting approximately 1.5 min. Slices were acquired coronally with the following parameters: turbo spin echo, 800 μm × 800 μm in-plane resolution, slice thickness = 2 mm or 1 mm, TR = 3000 ms, TE = 22 or 26 ms, 256 × 256 matrix, and flip angle = 120°.

**Fig. 3:**
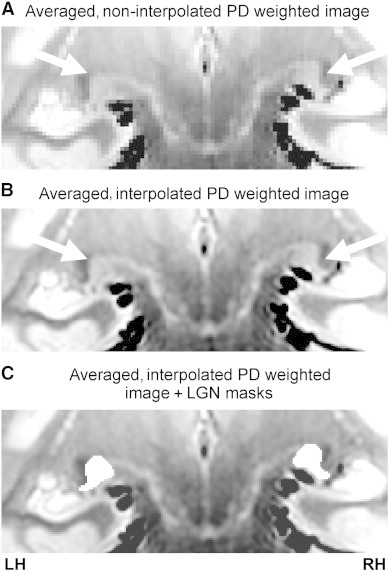
Coronal slice of an averaged PD weighted image depicting the LGN (white arrows) of a control participant when the PD images were (A) not interpolated and when they were (B) interpolated. Left and right ROI tracings of the LGN (white) in the averaged, interpolated PD weighted image are also shown (C).

Processing of PD weighted images was also conducted using FSL's toolbox. All PD weighted images for each participant were first interpolated to twice the resolution and half the voxel size to create a higher resolution image with FLIRT (Jenkinson and Smith, 2001; Jenkinson et al., 2002). To increase the signal-to-noise ratio (SNR), these interpolated images were then concatenated using fslmerge, motion corrected using MCFLIRT (Jenkinson et al., 2002), and a mean high resolution image was created using fslmaths (Fig. 3). Three independent raters manually traced left and right LGN ROI masks three times each per participant using the mean PD weighted image. For each rater, ROIs were merged using fslmerge and a median mask was created using fslmaths. Finally, median masks from each of the three raters were merged together and a final median mask across raters was created. LGN volumes (left and right) were calculated per participant from this final median mask using fslstats.

The methods used in this study are the gold standard and most appropriate methods for evaluating the chiasm and LGN in clinical settings (e.g. Bridge et al., 2009; Schmitz et al., 2003). While contrasts on the T_1_ and PD weighted images may be sensitive to other factors such as extracellular space, gliosis, and axon diameter (e.g. Sloper et al., 1987) that may affect the quantitative interpretation of our findings, the measurements in our study are consistent with those found using histological or post mortem measurements (e.g. Andrews et al., 1997; Horton, 1997).

## 3 Results

### 3.1 Whole brain volume

No significant difference in whole brain volume was found for the early ME compared to control group, *t*(38) = − 0.90, *P* = 0.373, and the whole brain volume of both groups was within the range previously reported for healthy controls (Allen et al., 2002; Ge et al., 2002) (Table 2). Therefore, whole brain volume was not factored into our analyses.

### 3.2 Intra- and inter-rater reliabilities

Intraclass correlation coefficient (ICC) reliability analyses were conducted to ensure consistent measurements within (intra) and between (inter) raters. For chiasm measurements, all intra-rater ICCs were above 0.71 and all inter-rater ICCs were above 0.79. For LGN volume, all intra-rater ICCs were above 0.98 and all inter-rater ICCs were above 0.87. ICCs above 0.7 are considered to reflect a strong agreement between measures (Cohen, 2001); thus our ICCs indicate that measurements were consistent both within and between raters.

### 3.3 Optic chiasm measurements

Optic chiasm measurements were within the range previously reported for healthy controls (Parravano et al., 1993; Wagner et al., 1997; Schmitz et al., 2003). Consistent with other research (Wagner et al., 1997), no significant sex differences in optic chiasm width (X and Y planes) or volume were found for the control group (*P*s ≥ 0.264).

A 2 × 3 Analysis of Variance (ANOVA) with age as a covariate, group (control, early ME) as the between-groups variable, and chiasm measurement (volume, X plane width, Y plane width) as the within-groups variable revealed a significant group × chiasm measurement interaction, *F*(1,37) = 68.92, *P* < 0.001. A significant main effect of group was also found, *F*(1,37) = 68.99, *P* < 0.001.

**Fig. 4:**
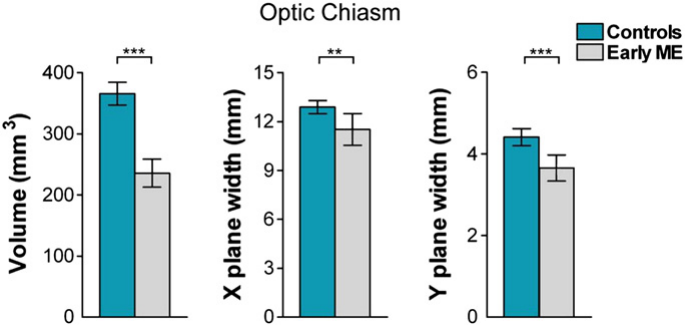
Bar graphs depicting mean optic chiasm volume (mm^3^) and mean optic chiasm width (mm) in the X and Y planes, for the control (blue bars) and early ME (grey bars) groups. The early ME group exhibited significant decreases in all chiasm measures compared to the control group. Error bars represent ± 95% confidence intervals (CIs). ^⁎⁎^*P* < 0.01; ^⁎⁎⁎^*P* < 0.001.

Posthoc pairwise comparisons (Bonferroni-adjusted alphas = 0.017) revealed that the early ME group had significantly decreased chiasm volume, and chiasm widths in the X and Y planes compared to the control group (*P*s ≤ 0.002). Fig. 4 shows mean optic chiasm X and Y plane widths and chiasm volume per group.

### 3.4 Optic nerve and optic tract diameter

No significant difference in diameter between left and right optic nerves, and left and right optic tracts, was found in the control group (*P*s ≥ 0.570) and these diameters were within the range previously reported for healthy controls (Parravano et al., 1993; Wagner et al., 1997; Schmitz et al., 2003). Consistent with other research (Jonas et al., 1992), no significant sex differences in optic nerve and optic tract diameter were found for the control group (*P*s ≥ 0.354). See Inline Supplementary Table S1 for information regarding the left and right optic nerves and tracts of the control group.

No significant difference in diameter between left and right optic nerves, and left and right optic tracts, was found in the control group (*P*s ≥ 0.570) and these diameters were within the range previously reported for healthy controls (Parravano et al., 1993; Wagner et al., 1997; Schmitz et al., 2003). Consistent with other research (Jonas et al., 1992), no significant sex differences in optic nerve and optic tract diameter were found for the control group (*P*s ≥ 0.354). See Inline Supplementary Table S1 for information regarding the left and right optic nerves and tracts of the control group.

Inline Supplementary Table S1

**Table S1:** Mean (± 95% CIs) left and right optic nerves and optic tract diameter, and LGN volume in the control group.

	Optic nerve diameter (mm)	Optic tract diameter (mm)	LGN volume (mm^3^)
Left	4.7 (0.2)	4.2 (0.1)	155 (18)
Right	4.8 (0.2)	4.3 (0.2)	164 (18)

Inline Supplementary Table S1

Inline Supplementary Table S1 can be found online at http://dx.doi.org/10.1016/j.nicl.2013.10.014.

A 2 × 2 × 2 ANOVA with age as a covariate, group (control, early ME) as the between-groups variable, and side (ipsilateral, contralateral) and diameter (optic nerve, optic chiasm) as the within-groups variables revealed a significant group × side × diameter interaction, *F*(1,37) = 36.01, *P* < 0.001. A trend towards a diameter × group interaction approached significance, *F*(1,37) = 3.39, *P* = 0.074. Significant main effects of diameter, *F*(1,37) = 11.69, *P* = 0.002, and group, *F*(1,37) = 44.08, *P* < 0.001, were also found.

**Fig. 5:**
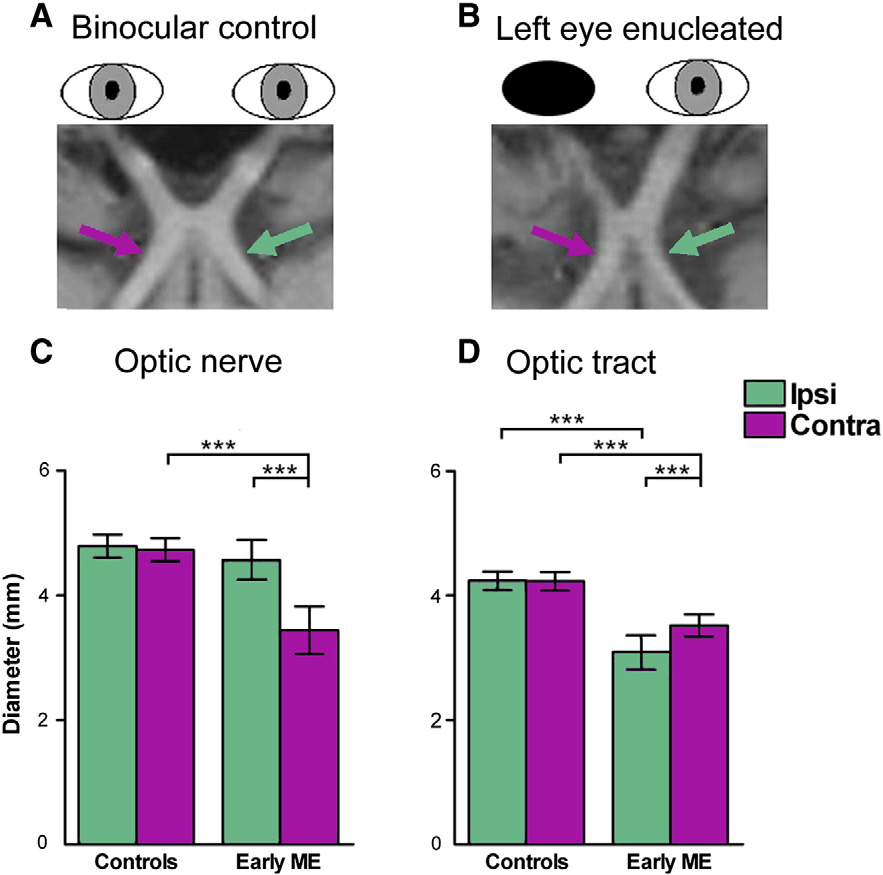
The optic nerves, chiasm, and tracts of a (A) typical control and (B) early ME participant are shown in reformatted T_1_ weighted images. Bar graphs are also shown depicting the mean diameter (mm) for (C) optic nerves and (D) optic tracts ipsilateral (Ipsi: green bars) and contralateral (Contra: purple bars) to the dominant eye in the control group and remaining eye in the early ME group. The early ME group exhibited significantly decreased diameter for the contralateral optic nerve and both optic tracts compared to the control group. The early ME group also exhibited an asymmetry that was not present in the control group: the contralateral optic tract (purple arrow) was significantly increased compared to the ipsilateral optic tract (green arrow) relative to the remaining eye. Error bars represent ± 95% CIs. ^⁎⁎⁎^*P* < 0.001.

Posthoc pairwise comparisons (Bonferroni-adjusted alphas = 0.013) revealed that the early ME group had significantly decreased contralateral optic nerve, and contralateral and ipsilateral optic tract diameters compared to the control group (*P*s < 0.001). There was no significant difference for ipsilateral optic nerve (*P* = 0.271) between groups. The early ME group exhibited asymmetries (Bonferroni-adjusted alphas = 0.025) where the contralateral optic nerve diameter was significantly decreased relative to the ipsilateral optic nerve, and the contralateral optic tract diameter was significantly increased relative to the ipsilateral optic tract (*P*s < 0.001). No significant asymmetries in optic nerve or tract diameter were found for the control group (*P*s ≥ 0.569). Fig. 5 shows optic chiasms from (A) typical control and (B) early ME participants, and bar graphs for mean (C) optic nerve and (D) optic tract diameter per group.

### 3.5 LGN volume

No significant difference in volume between left LGN and right LGN was found for the control group (*P* = 0.200), and these volumes were within the range previously reported for healthy controls (Putnam, 1926; Andrews et al., 1997; Dai et al., 2011). Consistent with other research (Li et al., 2012), no significant sex difference in LGN volume were found for the control group (*P*s ≥ 0.322).

A 2 × 2 ANOVA with age as a covariate, group (control, early ME) as the between-groups variable and side (ipsilateral, contralateral) as the within-groups variable revealed a significant group × side interaction, *F*(1,20) = 5.56, *P* = 0.029, and a significant main effect of group, *F*(1,20) = 14.21, *P* = 0.001. No significant main effect of side was found, *F*(1,20) = 0.35, *P* = 0.558.

**Fig. 6:**
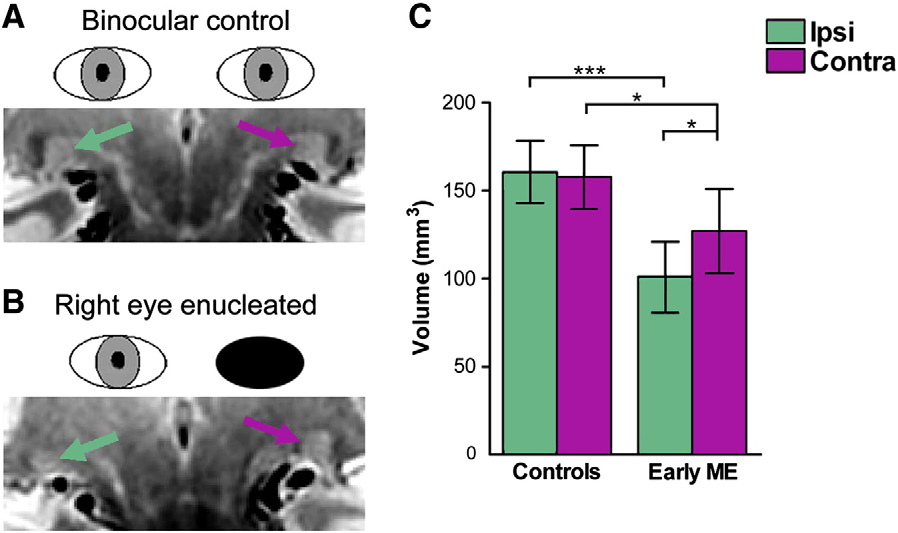
PD weighted images depicting the LGN ipsilateral (green arrow) and contralateral (purple arrow) to the dominant eye for a (A) typical control and remaining eye in a (B) typical early ME participant. A bar graph is shown (C) depicting mean LGN volumes (mm^3^) ipsilateral (Ipsi: green bars) and contralateral (Contra: purple bars) to the dominant eye in the control group and remaining eye in the early ME group. Compared to the control group, the early ME group exhibited volume decreases in both LGN. The early ME group also exhibited an asymmetry that was not present in the control group: contralateral LGN volume was significantly increased compared to ipsilateral LGN relative to the remaining eye. Error bars represent ± 95% CIs. ^⁎^*P* < 0.05; ^⁎⁎⁎^*P* < 0.001.

Posthoc pairwise comparisons (Bonferroni-adjusted alphas = 0.025) revealed that compared to the control group, the early ME group had significantly decreased ipsilateral LGN volume (*P* < 0.001) and a tendency for significantly decreased contralateral LGN volume (*P* = 0.045) that approached significance. Further posthoc pairwise comparisons (Bonferroni-adjusted alphas = 0.025) revealed that the early ME group exhibited a significant asymmetry with a decrease in ipsilateral compared to contralateral LGN volume (*P* = 0.017). The control group did not exhibit this asymmetry (*P* = 0.658). Fig. 6 shows PD weighted images of (A) typical control and (B) early ME participants, as well as a bar graph for mean LGN volume per group (C) (see Inline Supplementary Table S2 for percent differences in the early ME group compared to controls for optic nerve, chiasm, and tract measurements and LGN volume).

Posthoc pairwise comparisons (Bonferroni-adjusted alphas = 0.025) revealed that compared to the control group, the early ME group had significantly decreased ipsilateral LGN volume (*P* < 0.001) and a tendency for significantly decreased contralateral LGN volume (*P* = 0.045) that approached significance. Further posthoc pairwise comparisons (Bonferroni-adjusted alphas = 0.025) revealed that the early ME group exhibited a significant asymmetry with a decrease in ipsilateral compared to contralateral LGN volume (*P* = 0.017). The control group did not exhibit this asymmetry (*P* = 0.658). Fig. 6 shows PD weighted images of (A) typical control and (B) early ME participants, as well as a bar graph for mean LGN volume per group (C) (see Inline Supplementary Table S2 for percent differences in the early ME group compared to controls for optic nerve, chiasm, and tract measurements and LGN volume).

Inline Supplementary Table S2

**Table S2:** Percent (%) change in each chiasm structure and LGN volume for: the early ME group relative to the control group; the late ME participant relative to the control group and to age-matched controls; the late ME participant relative to the early ME group and to age-matched early ME participants. Percent change was calculated using the following formula: % change = (Comparison − Target) / Comparison × 100. Positive numbers indicate a decrease, and negative numbers indicate an increase. E.g. Percent change for early ME group (Target) relative to control group (Comparison) = (Control − Early ME) / Control × 100.

Target:comparison	Optic nerve diameter (mm)		Optic chiasm width (mm)		Optic chiasm volume (mm^3^)	Optic tract diameter (mm)		LGN volume (mm^3^)	
	Ipsi^α^	Contra	X plane	Y plane		Ipsi	Contra	Ipsi	Contra
Early ME:controls	4%	28%	11%	16%	35%	26%	17%	37%	19%
Late ME:controls	− 10%	13%	9%	16%	16%	19%	− 2%	49%	50%
Late ME:matched controls	3%	26%	20%	28%	21%	26%	− 5%	45%	55%
Late ME:early ME	− 15%	21%	− 2%	0%	− 30%	− 10%	− 23%	20%	39%
Late ME:matched early ME	− 13%	− 11%	8%	− 2%	− 51%	3%	− 9%	N/A	N/A

Inline Supplementary Table S2

Inline Supplementary Table S2 can be found online at http://dx.doi.org/10.1016/j.nicl.2013.10.014.

Upon exploration of the data, only one early ME participant (ME05) showed the opposite asymmetry pattern where the ipsilateral LGN was larger compared to the contralateral LGN. When this participant was removed from the analyses, we see no significant group difference for the contralateral LGN (*P* = 0.10), and an even larger within group difference for the early ME group where the contralateral LGN (Mean = 133 mm^3^, SD = 26 mm^3^) was significantly smaller than the ipsilateral LGN (Mean = 98 mm^3^, SD = 24 mm^3^) (*P* = 0.003). We could not find any reason to account for this participant's differences from other early ME participants (i.e. no cognitive deficits or neural disease, no visual abnormalities in remaining eye) and individual variability in LGN size and shape may be a contributing factor (Hickey and Guillery, 1979; Andrews et al., 1997).

### 3.6 Timing of deprivation

Partial correlations controlling for age (uncorrected alpha) revealed a significant positive correlation between AAE in the early ME group and the enucleated eye's optic nerve morphology such that earlier eye removal was associated with smaller optic nerve width, *r*(9) = 0.63, *P* = 0.039. However, the participant enucleated at 48 months of age was an outlier, and this correlation became non-significant after this participant was removed from the analysis, *r*(8) = 0.26, *P* = 0.474. Therefore, no significant relationship with AAE was found for any of the chiasm measurements (*P*s ≥ 0.177) or for LGN volume (*P*s ≥ 0.600).

### 3.7 Late ME

**Fig. 7:**
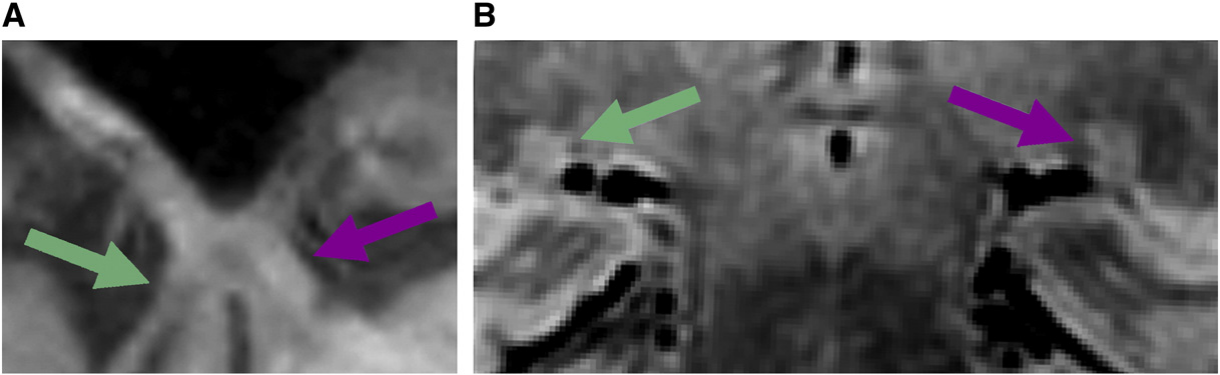
A T_1_ weighted image showing the (A) optic nerves, chiasm, and tracts, and (B) an averaged PD image showing both LGN from late ME (right eye enucleated) participant. The chiasm and LGN were decreased in size compared to controls. While the late ME showed an increase in contralateral (purple arrow) compared to ipsilateral (green arrow) optic tract diameter, there was no asymmetry between ipsilateral and contralateral LGN volumes.

We gathered morphological data from one late ME participant (Table 2 and Fig. 7). We used a modified *t*-test (Crawford and Garthwaite, 2002) designed to compare one individual case (i.e. the late ME participant) to a group of individuals (i.e. control and early ME groups). Table 2 shows chiasm measurements and LGN volume for the late ME participant. See Inline Supplementary Table S2 for percent differences in chiasm and LGN volume in the late ME participant relative to the control group and to the early ME group. Percent difference for each chiasm measurement and LGN volume were calculated using the following formula: % difference = (Comparison − late ME)/Comparison × 100.

We gathered morphological data from one late ME participant (Table 2 and Fig. 7). We used a modified *t*-test (Crawford and Garthwaite, 2002) designed to compare one individual case (i.e. the late ME participant) to a group of individuals (i.e. control and early ME groups). Table 2 shows chiasm measurements and LGN volume for the late ME participant. See Inline Supplementary Table S2 for percent differences in chiasm and LGN volume in the late ME participant relative to the control group and to the early ME group. Percent difference for each chiasm measurement and LGN volume were calculated using the following formula: % difference = (Comparison − late ME)/Comparison × 100.

#### 3.7.1 Late ME versus control group

Compared to the control group, the late ME participant exhibited decreased ipsilateral optic tract diameter, *t*(27) = − 2.04, *P* (one-tailed) < 0.050. No other differences between the late ME participant and control group were found for any of the remaining chiasm measurements. The late ME participant exhibited significantly decreased volume for both ipsilateral, *t*(7) = − 2.55, *P* (one-tailed) < 0.025, and contralateral, *t*(7) = − 2.44, *P* (one-tailed) < 0.025, LGN compared to the control group. When the late ME participant was compared to age- and sex-matched controls, the same pattern of results was found, with the addition of further decreases in chiasm width in the Y plane, *t*(1) = − 3.55, *P* (one-tailed) < 0.05, and in chiasm volume, *t*(1) = − 7.27, *P* (one-tailed) < 0.05. However, only two age-matched controls were available for chiasm measurements, and one for LGN volume suggesting this comparison should be interpreted with caution.

#### 3.7.2 Late ME versus early ME group

Compared to the early ME group, the late ME participant exhibited increased contralateral optic tract diameter, *t*(11) = 2.90, *P* (one-tailed) < 0.01, and increased chiasm volume, *t*(11) = 2.07, *P* (one-tailed) < 0.05. No other difference between the late ME participant and early ME group was found. The same pattern of results was found for chiasm measurements when the late ME was compared to an age- and sex-matched early ME participant, although, no matched early ME participant was available for LGN volume comparison. While the late ME participant did exhibit the pattern of increased contralateral compared to ipsilateral optic tract diameter, this participant did not exhibit the LGN asymmetry found in the early ME group.

## 4 Discussion

Compared to binocularly intact controls, we found morphological changes in the adult anterior visual system subsequent to early monocular enucleation due to retinoblastoma. Consistent with previous research (Goldby, 1957; Hickey and Guillery, 1979; Beatty et al., 1982; Horton, 1997), the early ME group exhibited significant degeneration of this system, which is expected given that half of visual inputs to the brain are removed during development. Surprisingly, however, we found an asymmetry in morphology where optic tracts and LGN decreases were less severe contralateral to the remaining eye. This novel finding has not previously been reported by enucleated monkey and human studies (Goldby, 1957; Matthews, 1964; Hubel et al., 1977; Rakic, 1981; Beatty et al., 1982; Hendrickson and Tigges, 1985; Sloper et al., 1987; Horton, 1997), which may reflect the failure of these other studies to assess long-term survival following eye enucleation that occurs early in life. This notion is supported by the large LGN volume decreases but a lack of asymmetry in contralateral/ipsilateral LGN volume in our late enucleated participant, coupled with marked geniculate cell degeneration in previous late enucleation studies (e.g. Goldby, 1957; Beatty et al., 1982). Our findings suggest that the complex postnatal organization of the visual system is altered following monocular enucleation that occurs during postnatal critical periods when the brain is still developing. Our data also suggest that although the anterior visual system is morphologically adult-like by 9 months of age (de Courten and Garey, 1982; Garey and de Courten, 1983), its critical developmental period extends beyond this age.

### 4.1 Morphological asymmetry

The optic tract and LGN morphological asymmetry observed in our study with early monocular enucleation is consistent with stronger functional activation in primary visual cortex (V1) contralateral to the remaining eye of anaesthetized early enucleated children (Barb et al., 2011), and in contralateral LGN and V1 of monocular viewing controls during ipsilateral eye stimulation (Miki et al., 2001; Toosy et al., 2001). The asymmetry is also consistent with morphological studies of enucleated rodents and rabbits that exhibit a contralateral bias in retinal projections and LGN size (Godement et al., 1987; Guillery, 1989; Khan, 2005). It is speculated that the disparity in previous results between enucleated primates and rodents is due to the difference in spatial layout of the chiasm during development. In binocularly intact rodents, retinal fibres mingle within the chiasm prior to projecting to either hemisphere. Axon guidance is considered to rely on this mingling and abnormal projections occur when retinal fibres from one eye are lacking (Neveu et al., 2006). In primates, enucleation is thought to have no impact on the remaining eye's projections since retinal projections are spatially segregated and no mingling occurs (Taylor and Guillery, 1995; Jeffery et al., 2008). Our data, however, do not support this theory since we do see asymmetries comparable to those found in rodents. Our findings therefore indicate the importance of surviving past the developmental stage in order to observe effects that early enucleation may have on the visual system. Possible explanations of this altered development must be explored.

### 4.2 Possible mechanisms of altered visual development

Normal visual development depends on the presence of activity-driven binocular interactions during postnatal maturation that are lacking with monocular enucleation (Sloper, 1993). For example, segregation of eye-specific laminae in the LGN and V1 relies on balanced binocular competition for space. Subsequent cell maintenance relies on binocular cooperation, but enucleation eliminates this cooperation and results in geniculate cell shrinkage (Sloper et al., 1987; Sloper, 1993). The LGN asymmetry found in our study may reflect changes in response to the loss of these binocular interactions. Here, we posit several possible mechanisms of altered visual development. First, aberrant connections may be formed between the remaining eye and geniculate cells associated with the enucleated eye, a phenomenon that has been observed in enucleated monkeys (Rakic, 1981). The contralateral bias in the optic tracts of our early ME participants, as well as that previously seen for enucleated rodents (Godement et al., 1987; Guillery, 1989), suggests that the crossed nasal fibres may be behind this cell recruitment. In line with this are behavioural data showing a preference for nasalward over temporalward motion for eye movement responses, specifically optokinetic nystagmus (OKN) (Reed et al., 1991; Day, 1995), and for coherent motion discrimination (Steeves et al., 2002) in early monocularly enucleated patients. Further supporting this notion are behavioural findings of earlier development of peripheral acuity in the temporal visual field (i.e. nasal retina) compared to nasal visual field (i.e. temporal retina) (Lewis and Maurer, 1992; Courage and Adams, 1996), and morphological data showing higher cone density in the nasal compared to temporal retina in adults (Curcio et al., 1990). Evidence against this notion stems from the prenatal establishment of retinal projections and laminar distribution of geniculate cells (Rakic, 1976; de Courten and Garey, 1982; Blakemore and Vital-Durand, 1986), and postnatally equivalent growth rates of geniculate cells in layers receiving crossed and uncrossed inputs in monkeys (Gottlieb et al., 1985). The late ME participant in our study exhibited an optic tract asymmetry comparable to that found for the early ME group, but no LGN asymmetry since volume in both LGN was decreased by about half compared to controls. Hence, the recruitment of geniculate cells via crossing nasal fibres may not be the only factor contributing to the LGN asymmetry found in our early ME group and additional factors must be considered. For example, it is also possible that optic tract asymmetries reflect, in part, altered development of the retinotectal pathway, as seen in early enucleated rodents (Woo et al., 1985). Further, neurons within parvocellular laminae of monkeys are more adversely affected by early monocular enucleation compared to neurons in magnocellular laminae (Sloper et al., 1987). This discrepancy may account for some of the observed volume asymmetry in the LGN, particularly given that parvocellular layers comprise a larger proportion of the LGN.

Another plausible mechanism for the observed asymmetry is that feedback connections from cortical area V1 are promoting the retention of deafferented geniculate cells during development, and this retention is stronger in the hemisphere contralateral to the remaining eye. Support for feedback from V1 stems from data showing the presence of binocular rivalry within the LGN of healthy controls (Wunderlich et al., 2005), and by retrograde degeneration of the right, but not left, LGN following right occipital lobe removal (Beatty et al., 1982). Geniculate axons pass through the optic radiation to synapse with neurons in layer IV of V1 and form ocular dominance columns (ODCs) — alternating columns of cells that are eye-specific. Binocularly intact monkeys have a greater contribution of crossed nasal retinal inputs to ODCs (Tychsen and Burkhalter, 1997) and monocular viewing controls and early enucleated children exhibit a contralateral bias in functional activity in V1 (Toosy et al., 2001; Barb et al., 2011). Together, these findings suggest that V1 receives stronger physiological inputs from the contralateral eye. Similar to geniculate laminae, ODCs require binocular interactions such as competition to develop in a normal fashion; however, they are only partially segregated at birth (Rakic, 1976; Sloper, 1993; Horton and Hocking, 1998). Since monocular enucleation completely obliterates ODCs in monkeys and humans (Rakic, 1981; Horton and Hocking, 1998), the remaining eye may be recruiting deafferented space within V1 while it is still maturing. A greater portion of visual cortex devoted to the remaining eye, particularly in the contralateral hemisphere, would translate into a stronger feedback signal to the LGN and cell preservation could occur predominantly in the LGN contralateral to the remaining eye. Consequently, some retinal fibres, especially the crossed nasal fibres, would also be preserved.

Alternatively, it could be argued that the observed LGN asymmetry solely reflects disproportionate neuronal degeneration due to the slightly unequal distribution of crossed versus uncrossed (~ 58:42) fibres found in the human and non-human primate visual systems (Chacko, 1948; Horton, 1997). However, the morphological decrease in the early ME group relative to the control group does not reflect this slight proportional asymmetry — decreases were much less than 50%, which would not be expected if neuronal degeneration was the sole determinant of changes in this population. While contralateral reductions were mild relative to the control group (optic tract — 17%; LGN — 19%), ipsilateral reductions were much more severe in the early ME group (optic tract — 26%; LGN — 37%) (Inline Supplementary Table S2). For example, when comparing to controls, volume decrease in the ipsilateral LGN of the early ME participants was almost double compared to that found in the contralateral LGN. Asymmetries should also be reflected with late monocular enucleation, yet an asymmetry has not been observed in previous late enucleation studies, nor in our late ME participant who exhibited large volume decreases in both LGN (~ 50%) compared to controls and in the contralateral LGN (~ 40%) compared to the early ME group. One study found a ratio of 53:47 for crossed versus uncrossed projections in a late enucleated man (Kupfer et al., 1967), which is less asymmetrical than that found for binocularly intact human and non-human primates (~ 58:42) (Chacko, 1948; Horton, 1997). Lastly, anophthalmic patients born with only one eye exhibit symmetrical visual evoked potentials (VEPs) in visual cortex, reflecting an equal decussation of retinal fibres at the chiasm (Neveu et al., 2006). Therefore, an unequal number of nasal versus temporal retinal projections to the visual system cannot completely account for the asymmetries observed in our study. It is more likely that the asymmetry reflects reorganization during development via aberrant synaptic connections and/or feedback from contralateral V1 to retain deafferented geniculate cells.

Alternatively, it could be argued that the observed LGN asymmetry solely reflects disproportionate neuronal degeneration due to the slightly unequal distribution of crossed versus uncrossed (~ 58:42) fibres found in the human and non-human primate visual systems (Chacko, 1948; Horton, 1997). However, the morphological decrease in the early ME group relative to the control group does not reflect this slight proportional asymmetry — decreases were much less than 50%, which would not be expected if neuronal degeneration was the sole determinant of changes in this population. While contralateral reductions were mild relative to the control group (optic tract — 17%; LGN — 19%), ipsilateral reductions were much more severe in the early ME group (optic tract — 26%; LGN — 37%) (Inline Supplementary Table S2). For example, when comparing to controls, volume decrease in the ipsilateral LGN of the early ME participants was almost double compared to that found in the contralateral LGN. Asymmetries should also be reflected with late monocular enucleation, yet an asymmetry has not been observed in previous late enucleation studies, nor in our late ME participant who exhibited large volume decreases in both LGN (~ 50%) compared to controls and in the contralateral LGN (~ 40%) compared to the early ME group. One study found a ratio of 53:47 for crossed versus uncrossed projections in a late enucleated man (Kupfer et al., 1967), which is less asymmetrical than that found for binocularly intact human and non-human primates (~ 58:42) (Chacko, 1948; Horton, 1997). Lastly, anophthalmic patients born with only one eye exhibit symmetrical visual evoked potentials (VEPs) in visual cortex, reflecting an equal decussation of retinal fibres at the chiasm (Neveu et al., 2006). Therefore, an unequal number of nasal versus temporal retinal projections to the visual system cannot completely account for the asymmetries observed in our study. It is more likely that the asymmetry reflects reorganization during development via aberrant synaptic connections and/or feedback from contralateral V1 to retain deafferented geniculate cells.

### 4.3 Anterior visual system degeneration

Although altered visual development was apparent in our early ME group, relative decreases were nonetheless present in both chiasm and LGN measures compared to binocularly intact controls. Wallerian degeneration of axons can occur when the optic nerve is severed and explains the decreases in chiasm structures found with our early ME group and late ME participant. However, the mean diameter of the enucleated eye's optic nerve was not even decreased by half, which suggests the presence of Wilbrand's knee — an artefact of enucleation where crossed fibres of the remaining eye loop into the contralateral optic nerve before heading to the chiasm and tract. This phenomenon may be caused by the shrinkage of the optic chiasm (Horton, 1997).

While we cannot say with certainty that decreases observed in MRI volumes and diameters represent an actual decrease in neuron counts, neuron size, or smaller versus fewer fibres, we know that the total chiasm diameters are smaller and total LGN volumes are smaller. As mentioned earlier, maintenance of cells within the LGN relies on activity-driven cooperation between the two eyes (Sloper et al., 1987; Sloper, 1993). Since the LGN is well developed at birth (de Courten and Garey, 1982; Garey and de Courten, 1983), decreases in the LGN of our early ME participants likely reflect a decrease in neuron size (e.g. Sloper et al., 1987), and/or transneuronal degeneration as a result of the loss of synaptic input and binocular cooperation (e.g. Goldby, 1957). Unbalanced binocular cooperation has been shown to disrupt LGN development in other forms of monocular deprivation such as amblyopia where the quality of visual input from both eyes is unequal (e.g. Barnes et al., 2010). Degeneration was also found with the late ME participant in our study, and at least for the LGN, these decreases were larger than the early ME group. Degeneration of the anterior visual system occurs with visual deprivation at any age; however, it is clear that this degeneration is less severe with earlier ME suggesting altered development that may be compensating for early eye loss.

## 5 Conclusion

In conclusion, we examined morphological development of the anterior visual system in a group of adults who had one eye enucleated early in life due to retinoblastoma. In contrast to previous research, we observed an asymmetry in morphology where decreases were less severe in the optic tracts and LGN contralateral to the remaining eye. This novel finding indicates a possible recruitment of deafferented geniculate cells via the crossing nasal fibres and/or stronger feedback signals from contralateral cortical area V1 that aids in the retention of deafferented cells. Altered visual system development may help explain the lack of spatial vision deficits in this population. In line with previous notions of plasticity and consistent with previous research on late enucleation, a lack of asymmetry but substantial decrease in volume both in geniculate nuclei in the late enucleated participant indicates stronger malleability of the brain following visual deprivation that occurs earlier rather than later in life. These data highlight the importance of balanced binocular input during postnatal maturation for anterior visual system morphology. Future studies should expand on these data to determine whether visual areas and white matter integrity within the visual cortex are also affected by early monocular enucleation in humans.

## Funding

This research was funded by the Natural Sciences and Engineering Research Council of Canada, the Canada Foundation for Innovation, and the Canadian National Institute for the Blind. Publication was made possible by the Open Access Author's Fund provided by York University.

## Figures and Tables

Figure adapted from ©John Wiley and Sons, Inc.

**Table 1:** ME patient histories. All participants took part in the chiasm phase of the experiment. Participants that took part in the LGN phase are indicated under LGN volume. Age, sex, Snellen acuity, enucleated eye, and age at enucleation (AAE) are also reported.

Patient	Age (years)^a^	Sex	Acuity	Enucleated eye	AAE (months)	LGN volume^b^
ME01	54	Male	20/16 − 1	Right	24	No
ME02	43/45	Female	20/12.5 + 2	Right	18	Yes
ME03	21	Male	20/20	Right	23	No
ME04	18	Female	20/20	Right	48	No
ME05	18/19	Male	20/20 + 4	Right	13	Yes
ME06	17	Male	20/20 + 2	Right	9	No
ME07	29	Female	20/20	Left	11	Yes
ME08	28	Male	20/16	Right	4	Yes
ME09	30	Male	20/20 + 3	Left	13	Yes
ME10	18	Female	20/16 + 3	Right	17	Yes
ME11	17	Female	20/12.5 + 1	Right	26	Yes
ME12	36	Female	20/16 + 4	Left	18	Yes
ME13	65	Male	20/20	Right	708	Yes

a The first number indicates age for the chiasm phase, the second number indicates age for the LGN phase.

b Indicates whether participant took part in the LGN phase of the study.

**Table 2:** Descriptive statistics for the control and early ME groups showing the mean (± 95% CIs) optic nerve diameter, optic chiasm width in the X and Y planes, optic chiasm volume, optic tract diameter, LGN volume, and whole brain volume. Values for each measurement are also shown for one late ME participant.

	Optic nerve diameter (mm)		Optic chiasm width (mm)		Optic chiasm volume (mm^3^)	Optic tract diameter (mm)		LGN volume (mm^3^)		Whole brain volume (cm^3^)
Group	Ipsi^a^	Contra	X plane	Y plane		Ipsi	Contra	Ipsi	Contra	
Control	4.8 (0.2)	4.7 (0.2)	12.9 (0.4)	4.4 (0.2)	365 (18)	4.2 (0.2)	4.2 (0.2)	160 (18)	157 (18)	1098 (41)
Early ME	4.6 (0.3)	3.4 (0.4)^b^	11.5 (1.0)^b^	3.7 (0.3)^b^	236 (23)^b^	3.1 (0.3)^b^	3.5 (0.2)^b^	101 (20)^b^	127 (24)^b^	1130 (61)
Late ME	5.3	4.1	11.7	3.7	307^c^	3.4^b^	4.3^c^	81^b^	78^b^	1069

a Ipsilateral to the dominant eye for controls or the remaining eye for ME participants.

b Significantly different from the control group.

c Significantly different from the early ME group.
