# Supplementary material for: Altered anterior visual system development following early monocular enucleation
Source: Neuroimage Clin. 2013 Nov 1;4:72–81. doi: 10.1016/j.nicl.2013.10.014 (PMC3853349; doi:10.1016/j.nicl.2013.10.014)
Supplement: Inline Supplementary Table S1 [file mmc1.doc]

Supplementary Table 1. Mean (±95% CIs) left and right optic nerve

and optic tract diameter, and LGN volume in the control group.

|  | Optic nerve Optic tract LGN Volume  diameter (mm) diameter (mm) (mm^3^) | | |
| --- | --- | --- | --- |
| Left | 4.7(0.2) | 4.2(0.1) | 155(18) |
| Right | 4.8(0.2) | 4.3(0.2) | 164(18) |
