# Supplementary material for: Altered anterior visual system development following early monocular enucleation
Source: Neuroimage Clin. 2013 Nov 1;4:72–81. doi: 10.1016/j.nicl.2013.10.014 (PMC3853349; doi:10.1016/j.nicl.2013.10.014)
Supplement: Inline Supplementary Table S2 [file mmc2.docx]

Supplementary Table 2. Percent (%) difference in each chiasm structure and LGN volume for: the early ME group relative to the control group; the late ME participant relative to the control group and to age-matched controls; the late ME participant relative to the early ME group and to age-matched early ME participants. Percent difference was calculated using the following formula: % difference = (Comparison – Target)/Comparison × 100. Positive numbers indicate a decrease, and negative numbers indicate an increase. E.g. Percent difference for early ME group (Target) relative to control group (Comparison) = (Control – Early ME)/Control × 100.

|  | | Optic nerve Optic chiasm Optic chiasm Optic tract LGN Volume  diameter (mm) width (mm) volume (mm^3^) diameter (mm) (mm^3^) | | | | | | | | | | |
| --- | --- | --- | --- | --- | --- | --- | --- | --- | --- | --- | --- | --- |
| Target: Comparison |  | Ipsi^α^ | Contra |  | X Plane | Y Plane |  | Ipsi | Contra |  | Ipsi | Contra |
|  | |  |  |  |  |  |  |  |  |  |  |  |
| Early ME: Controls | | 4% | 28% |  | 11% | 16% | 35% | 26% | 17% |  | 37% | 19% |
| Late ME: Controls | | -10% | 13% |  | 9% | 16% | 16% | 19% | -2% |  | 49% | 50% |
| Late ME: Matched Controls | | 3% | 26% |  | 20% | 28% | 21% | 26% | -5% |  | 45% | 55% |
| Late ME: Early ME | | -15% | 21% |  | -2% | 0% | -30% | -10% | -23% |  | 20% | 39% |
| Late ME: Matched Early ME | | -13% | -11% |  | 8% | -2% | -51% | 3% | -9% |  | N/A | N/A |
